# Supplementary material for: The Predicted Splicing Variant c.11+5G>A in RPE65 Leads to a Reduction in mRNA Expression in a Cell-Specific Manner
Source: Cells. 2022 Nov 17;11(22):3640. doi: 10.3390/cells11223640 (PMC9688607; doi:10.3390/cells11223640)
Supplement: Supplementary file 1 [file cells-11-03640-s001.zip › cells-1971293-supplementary.pdf]

The Predicted Splicing Variant c.11+5G>A in *RPE65* Leads to a reduction in mRNA Expression in a Cell-Specific Manner

Irene Vázquez-Domínguez[1,2], Lonneke Duijkers[1], Zeinab Fadaie[1,2], Eef C.W. Alaerds[1], Merel A. Post[3], Edwin M. van Oosten[4,5], Luke O'Gorman[1], Michael Kwint[1], Louet Koolen[6], Anita D.M. Hoogendoorn[1], Hester Y. Kroes[7], Christian Gilissen[1,5], Frans P.M. Cremers[1,2], Rob W.J. Collin[1,2,#], Susanne Roosing[1,2,#] and Alejandro Garanto[1,4,5*].

[1]Department of Human Genetics, Radboud university medical center, 6525 GA, Nijmegen, The Netherlands.

[2]Donders Institute for Brain, Cognition and Behaviour, Radboud university medical center, G525 GD, Nijmegen, The Netherlands.

[3]Department of Neurology, Donders Institute for Brain, Cognition and Behaviour, Radboud university medical center, 6525GA, Nijmegen, The Netherlands.

[4]Department of Pediatrics, Amalia Children's hospital, Radboud university medical center, 6525 GA, Nijmegen, The Netherlands.

[5]Radboud Institute of Molecular Life Sciences (RIMLS), Radboud university medical center, 65265 GA Nijmegen, The Netherlands.

[6]Department of Ophthalmology, Radboud university medical center, 6525 GA, Nijmegen, The Netherlands.

[7]Division Laboratories, Pharmacy and Biomedical Genetics, Clinical Genetics, University Medical Center of Utrecht, 3584 CX, Utrecht, The Netherlands.

†These authors contributed equally to this work*Corresponding author

Supplementary material

Index

Supplementary tables. 2

Table S1. List of primers employed in this work. 2

Table S2. List and combinations of antibodies used for immunocytochemistry. 4

Table S3. List of antibodies used for Western blot analysis study 4

Table S4. Summary of the protein analysis of the ~35 kDa and 65 kDa protein band from the IP of RPE65. .. 5

Supplementary figures 8

Figure S1. Peptide coverage of RPE65 in for the 65 kDa band. 8

Figure S2. RT-PCR from exon 1 to 3 of the *RPE65* entry clones in different cell lines. 9

Supplementary tables.

Table S1. List of primers employed in this work.

Name	Sequence with tag/tails (5′-3′)	Sequence without tag/tails (5′-3′)	Tm(°C)	PCR product (bp)
**Splicing vectors**				
***RPE65*_5'UTR_Fwd**	GGGGACAAGTTTGTACAAAAAAGCAGGCTTCCAGAGAATGGTGCCAAGGTC	CAGAGAATGGTGCCAAGGTC	65.1	3,832
***RPE65*_intron3_Rev**	GGGGACCACTTTGTACAAGAAAGCTGGGTGTTGCCAAGGTTACCCTCCTA	TTGCCAAGGTTACCCTCCTA	63.7	
**SDMut_*RPE65*_c.11+5_Fwd**	n/a	CTGGAAGAAAATGTCTATCCAGTAAGTATCTCTGGGAGAC	73.8	3,832
**SDMut_*RPE65*_c.11+5_Rev**	n/a	CTGGAAGAAAATGTCTATCCAGTAAGTATCTCTGGGAGAC	73.8	
**Luciferase-reporter assay constructs**				
***RPE65*_XS_Intron 1.1_Fwd**	ACTGGAAGAAAATGTCTATCCAGTAACCTTGGCAACA	ACTGGAAGAAAATGTCTATCCA	58.0	5,508
***RPE65*_XS_5′UTR_Rev**	CCTGGGTGTTGCCAAGGTTACATTTATCATGAATCC	CCTGGGTGTTGCCAAGGTTAC	60.0	
***RPE65*_S_Intron 1_Fwd**	CACCTTCATGGATTCATGATAAATGTAACCTTGGCAACA	CACCTTCATGGATTCATGATAAAT	58.0	5,561
***RPE65*_S_5′UTR_Rev**	CCTGGGTGTTGCCAAGGTTACATTTATCATGAATCC	CCTGGGTGTTGCCAAGGTTAC	60.0	
***RPE65*_M _Intron 2.1_Fwd**	CGCTACCTAATTGGAATTCCCGTAACCTTGGCAACA	CGCTACCTAATTGGAATTCCC	60.0	6,992
***RPE65*_M_5′UTR_Rev**	CCTGGGTGTTGCCAAGGTTACATTTATCATGAATCC	CCTGGGTGTTGCCAAGGTTAC	60.0	
***RPE65*_L_Intron 2.2_Fwd**	ACTGTGAAAGCTGAGAGGCCGTAACCTTGGCAACA	ACTGTGAAAGCTGAGAGGCC	60.0	8,056
***RPE65*_L_5′UTR_Rev**	CCTGGGTGTTGCCAAGGTTACATTTATCATGAATCC	CCTGGGTGTTGCCAAGGTTAC	60.0	
**RT-PCR**				
***RPE65*_exon1_Fwd**	n/a	CTTCATTCTGCAGTTGGTGCC	62.0	164
***RPE65*_exon3_Rev**	n/a	CCACATCGAAGGAGACTGCC	58.0	
***ACTB*_exon3_Fwd**	n/a	ACTGGGACGACATGGAGAAG	60.5	398
***ACTB*_exon3_Rev**	n/a	TCTCAGCTGTGGTGGTGAAG	60.5	
**qPCR**				
***OCT3/4*_Fwd**	n/a	GTTCTTCATTCACTAAGGAAGG	58.4	101
***OCT3/4*_Rev**	n/a	CAAGAGCATCATTGAACTTCAC	58.4	

***PAX6*_Fwd**	n/a	GCTGCAAAGAAATAGAACATCC	58.4	111
***PAX6*_Rv**	n/a	TTGGCTGCTAGTCTTTCTCG	58.4	
***CRX*_Fwd**	n/a	CCCCAGTGTGGATCTGATG	59.5	116
***CRX*_Rv**	n/a	CAAACAGTGCCTCCAGCTC	59.5	
***OPN1SW*_Fwd**	n/a	TTCTTCTCCAAGAGTGCTTGC	59.5	97
***OPN1SW*_Rv**	n/a	CCTTCCCACACACCATCTTC	60.5	
***BEST1*_Fwd**	n/a	TCAGTGTGGACACCTGTATGC	61.2	84
***BEST1*_Rv**	n/a	AAGCTGTACACCGCCACAG	59.5	
***RPE65*_Fwd**	n/a	TTACTACGCTTGCACAGAGACC	62.1	105
***RPE65*_Rv**	n/a	GCCCCATTGACAGAGACATAG	61.2	
***MERTK*_Fwd**	n/a	TTGCAGCATTCAGGTCAAGGAAGC	65.2	106
***MERTK*_Rv**	n/a	GGCTTGCAGCTGCTTGATTTGGTA	65.2	
***DCT*_Fwd**	n/a	CTTTGCCGGCTATAATTGTG	56.4	101
***DCT*_Rv**	n/a	TCAAGGAATGGATGTTCTGC	56.4	
***ABCA4*_Fwd**	n/a	CATCCTGTTCCACCACCTCA	60.5	113
***ABCA4*_Rv**	n/a	CTGTGTCCTCCAACATGGCT	60.5	
***GUSB*_Fwd**	n/a	AGAGTGGTGCTGAGGATTGG	60.5	80
***GUSB*_Rv**	n/a	CCCTCATGCTCTAGCGTGTC	62.5	

Underlined sequences corresponded to tail/tag sequences. SDMut means side-directed mutagenesis. n/a means non-applicable for that primer.

Table S2. List and combinations of antibodies used for immunocytochemistry.

Gene	Antibody Catalog #	Manufacturer	Properties	Dilution
**Primary antibodies**				
**BEST1**	NB300-164	Novus Biologicals (CO, USA)	Monoclonal, Mouse	1:200
**EZRIN**	E8897	Sigma-Aldrich	Monoclonal, Mouse	1:250
**ZO-1**	40-2300	Invitrogen	Polyclonal, Rabbit	1:500
**RPE65**	MAB5428	Sigma-Aldrich	Monoclonal, Mouse	1:500
**Secondary antibodies**				
**Goat anti-rabbit 488**	A11008	Molecular Probes (ORE, USA)	Polyclonal	1:500
**Goat anti-mouse 568**	A11031	Life Technologies	Polyclonal	1:500
**Others**				
**DAPI**	0100-20	Southern Biotechnologies (AL, USA)		1:300 (of a 1:100 dilution)
**Combinations**				
**Antibody 1**	**Secondary Antibody 1**	**Antibody 2**	**Secondary antibody 2**	**DAPI**
**ZO-1**	Goat anti-rabbit 488	BEST1	Goat anti-mouse 568	Yes
**ZO-1**	Goat anti-rabbit 488	EZRIN	Goat anti-mouse 568	Yes
**ZO-1**	Goat anti-rabbit 488	RPE65	Goat anti-mouse 568	Yes

Table S3. List of antibodies used for Western blot analysis study

**Primary antibodies**	**Target protein**	**Producted by**	**Antibody Catalog #**	**Host**	**Diluent**	**WB dilution**
	RPE65	Sigma-Aldrich	MAB5428	Mouse (Monoclonal)	WestVisionTM block diluent	1:1000
	Histone H3	Abcam	ab32356	Rabbit (Monoclonal)	WestVisionTM block diluent	1:1000
	MERTK (Y323)	Abcam	ab52968	Rabbit (Monoclonal)	WestVisionTM block diluent	1:1000
**Secondary antibodies**	**Target host**	**Producted by**	**Antibody Catalog #**	**Host / Fluorophore**	**Diluent**	**WB dilution**
	Rabbit	Molecular Probes	A21076	Goat / Alexa Fluor 680	WestVisionTM block diluent	1:10000
	Mouse	Li-Cor Biosciences	926-32210	Goat / IRDye800	WestVisionTM block diluent	1:20000

Table S4. Summary of the protein analysis of the ~35 kDa and 65 kDa protein band from the IP of RPE65. The last two columns represent the total intensity of the detected peptide sequence from lower intensity (in red) to higher intensity (green), yellow colours indicate intermediate intensity. In bold, RPE65 protein which have an intensity affinity with the 65-kDa protein as expected. Proteins with coverage lower than 5.0, probability or top peptide probability lower than 0.999 and/or highest intensity for both bands lower than 50,000 were discarded during the filtrating process.

Protein ID	Entry Name	Gene	Protein Length	Coverage	Organism	Protein Existence	Protein Probability	Top Peptide Probability	35 Total Intensity	65 Total Intensity
O43852	CALU_HUMAN	CALU	315	10.8	Homo sapiens OX=9606	1:Experimental evidence at protein level	1	0.999	93,961.51	0
P00505	AATM_HUMAN	GOT2	430	24.2	Homo sapiens OX=9606	1:Experimental evidence at protein level	1	0.999	162,246.56	24,242.09
P02545	LMNA_HUMAN	LMNA	664	23.9	Homo sapiens OX=9606	1:Experimental evidence at protein level	1	0.999	0	105,971.945
P02768	ALBU_HUMAN	ALB	609	11.8	Homo sapiens OX=9606	1:Experimental evidence at protein level	1	0.999	2,501,452	5,493,824
P04406	G3P_HUMAN	GAPDH	335	13.1	Homo sapiens OX=9606	1:Experimental evidence at protein level	1	0.999	67,913.35	7,994.7446
P04843	RPN1_HUMAN	RPN1	607	11.5	Homo sapiens OX=9606	1:Experimental evidence at protein level	1	0.999	0	80,513.95
P10809	CH60_HUMAN	HSPD1	573	25	Homo sapiens OX=9606	1:Experimental evidence at protein level	1	0.999	0	113,916.625
P11166	GTR1_HUMAN	SLC2A1	492	12.4	Homo sapiens OX=9606	1:Experimental evidence at protein level	1	0.999	345,835	56,481.434
P29401	TKT_HUMAN	TKT	623	27.1	Homo sapiens OX=9606	1:Experimental evidence at protein level	1	0.999	0	203,020.9
P38646	GRP75_HUMAN	HSPA9	679	25.2	Homo sapiens OX=9606	1:Experimental evidence at protein level	1	0.999	0	279,579.03
P39656	OST48_HUMAN	DDOST	456	8.6	Homo sapiens OX=9606	1:Experimental evidence at protein level	1	0.999	57,738.64	0
Q07065	CKAP4_HUMAN	CKAP4	602	30.4	Homo sapiens OX=9606	1:Experimental evidence at protein level	1	0.999	0	133,184.78
Q9NPH2	INO1_HUMAN	ISYNA1	558	7	Homo sapiens OX=9606	1:Experimental evidence at protein level	1	0.999	0	67,233.82
A0A075B6S2	KVD29_HUMAN	IGKV2D-29	120	16.7	Homo sapiens OX=9606	3:Protein inferred from homology	1	0.999	700,050.06	128,539.875

O75874	IDHC_HUMAN	IDH1	414	10.1	Homo sapiens OX=9606	1:Experimental evidence at protein level	1	0.999	104,416.69	0
O94905	ERLN2_HUMAN	ERLIN2	339	14.2	Homo sapiens OX=9606	1:Experimental evidence at protein level	1	0.999	174,234.78	0
P00558	PGK1_HUMAN	PGK1	417	30.7	Homo sapiens OX=9606	1:Experimental evidence at protein level	1	0.999	164,836.4	0
P01859	IGHG2_HUMAN	IGHG2	326	16.3	Homo sapiens OX=9606	1:Experimental evidence at protein level	1	0.999	67,411.27	29,636.666
P01857	IGHG1_HUMAN	IGHG1	330	13.3	Homo sapiens OX=9606	1:Experimental evidence at protein level	1	0.999	86,852.52	79,244.375
P02790	HEMO_HUMAN	HPX	462	15.4	Homo sapiens OX=9606	1:Experimental evidence at protein level	1	0.999	0	193,785.7
P04075	ALDOA_HUMAN	ALDOA	364	38.5	Homo sapiens OX=9606	1:Experimental evidence at protein level	1	0.999	95,4295.2	0
P09972	ALDOC_HUMAN	ALDOC	364	8.2	Homo sapiens OX=9606	1:Experimental evidence at protein level	0.9995	0.999	117,448.11	0
P04264	K2C1_HUMAN	KRT1	644	55	Homo sapiens OX=9606	1:Experimental evidence at protein level	1	0.999	2,632,790.8	2,522,892.5
P35908	K22E_HUMAN	KRT2	639	51.2	Homo sapiens OX=9606	1:Experimental evidence at protein level	1	0.999	1,435,906	1,496,954
P13647	K2C5_HUMAN	KRT5	590	28.3	Homo sapiens OX=9606	1:Experimental evidence at protein level	1	0.999	986,652.44	560,298.8
P02538	K2C6A_HUMAN	KRT6A	564	30.1	Homo sapiens OX=9606	1:Experimental evidence at protein level	1	0.999	1,063,048	846,167.06
P04259	K2C6B_HUMAN	KRT6B	564	26.8	Homo sapiens OX=9606	1:Experimental evidence at protein level	1	0.999	1,531,210.5	1,464,059.1
P11142	HSP7C_HUMAN	HSPA8	646	39.9	Homo sapiens OX=9606	1:Experimental evidence at protein level	1	0.999	0	536,350.4
P0DMV8	HS71A_HUMAN	HSPA1A	641	21.8	Homo sapiens OX=9606	1:Experimental evidence at protein level	1	0.999	0	406,626.2
P13645	K1C10_HUMAN	KRT10	584	48.1	Homo sapiens OX=9606	1:Experimental evidence at protein level	1	0.999	1,595,172.6	1,333,126.6
P35527	K1C9_HUMAN	KRT9	623	43.7	Homo sapiens OX=9606	1:Experimental evidence at protein level	1	0.999	1,872,941.2	1,926,071.8
P08727	K1C19_HUMAN	KRT19	400	61	Homo sapiens OX=9606	1:Experimental evidence at protein level	1	0.999	984,447.1	415,205.28
P02533	K1C14_HUMAN	KRT14	472	44.5	Homo sapiens OX=9606	1:Experimental evidence at protein level	1	0.999	1,072,700	816,618.3
Q04695	K1C17_HUMAN	KRT17	432	24.8	Homo sapiens OX=9606	1:Experimental evidence at protein level	1	0.999	984,447.1	415,205.28

P08779	K1C16_HUMAN	KRT16	473	24.3	Homo sapiens OX=9606	1:Experimental evidence at protein level	1	0.999	1,072,700	816,618.3
P14618	KPYM_HUMAN	PKM	531	27.1	Homo sapiens OX=9606	1:Experimental evidence at protein level	1	0.999	0	254,464.88
P16403	H12_HUMAN	H1-2	213	25.4	Homo sapiens OX=9606	1:Experimental evidence at protein level	1	0.999	115,079.92	89,869.164
P10412	H14_HUMAN	H1-4	219	24.7	Homo sapiens OX=9606	1:Experimental evidence at protein level	1	0.999	135,708.25	82,180.445
P39023	RL3_HUMAN	RPL3	403	12.9	Homo sapiens OX=9606	1:Experimental evidence at protein level	1	0.999	109,001.164	3,278.821
P42025	ACTY_HUMAN	ACTR1B	376	9.6	Homo sapiens OX=9606	1:Experimental evidence at protein level	1	0.999	76,152.51	0
P63261	ACTG_HUMAN	ACTG1	375	77.9	Homo sapiens OX=9606	1:Experimental evidence at protein level	1	0.999	2.61E+07	177,051.66
P60709	ACTB_HUMAN	ACTB	375	77.9	Homo sapiens OX=9606	1:Experimental evidence at protein level	1	0.999	2.61E+07	177,051.66
P68032	ACTC_HUMAN	ACTC1	377	46.7	Homo sapiens OX=9606	1:Experimental evidence at protein level	1	0.999	2.51E+07	174,650.2
P61160	ARP2_HUMAN	ACTR2	394	21.1	Homo sapiens OX=9606	1:Experimental evidence at protein level	1	0.999	133,726.66	0
Q15019	SEPT2_HUMAN	SEPTIN2	361	12.5	Homo sapiens OX=9606	1:Experimental evidence at protein level	1	0.999	92,480.19	0
**Q16518**	**RPE65_HUMAN**	**RPE65**	**533**	**22.7**	**Homo sapiens OX=9606**	**1:Experimental evidence at protein level**	**1**	**0.999**	**4,680.633**	**904,031.3**
Q53GT1	KLH22_HUMAN	KLHL22	634	23.7	Homo sapiens OX=9606	1:Experimental evidence at protein level	1	0.999	0	196,319.53
Q9NYL9	TMOD3_HUMAN	TMOD3	352	44.6	Homo sapiens OX=9606	1:Experimental evidence at protein level	1	0.999	499,725.06	6,141.5503

Supplementary figures

```
        10         20         30         40         50         60         70
MSIQVEHPAG GYKKLFETVE ELSSPLTAHV TGRIPLWLTG SLLRCGPGLF EVGSEPFYHL FDGQALLHKF

        80         90        100        110        120        130        140
DFKEGHVTYH RRFIRTDAYV RAMTEKRIVI TEFGTCAFPD PCKNIFSRFF SYFRGVEVTD NALVNVYPVG

       150        160        170        180        190        200        210
EDYYACTETN FITKINPETL ETIKQVDLCN YVSVNGATAH PHIENDGTVY NIGNCFGKNF SIAYNIVKIP

       220        230        240        250        260        270        280
PLQADKEDPI SKSEIVVQFP CSDRFKPSYV HSFGLTPNYI VFVETPVKIN LFKFLSSWSL WGANYMDCFE

       290        300        310        320        330        340        350
SNETMGVWLH IADKKRKKYL NNKYRTSPFN LFHHINTYED NGFLIVDLCC WKGFEFVYNY LYLANLRENW

       360        370        380        390        400        410        420
EEVKKNARKA PQPEVRRYVL PLNIDKADTG KNLVTLPNTT ATAILCSDET IWLEPEVLFS GPRQAFEFPQ

       430        440        450        460        470        480        490
INYQKYCGKP YTYAYGLGLN HFVPDRLCKL NVKTKETWVW QEPDSYPSEP IFVSHPDALE EDDGVVLSVV

       500        510        520        530
VSPGAGQKPA YLLILNAKDL SEVARAEVEI NIPVTFHGLF KKS
```

Figure S1. Peptide coverage of RPE65 in for the 65 kDa band. Peptides found by mass spectrometry after in-gel digestion of immunoprecipitating the ~35 kDa and 65 kDa bands. In yellow, coverage of peptide sequences of the 65 kDa band mapped on RPE65 protein sequence obtained from Uniprot (Q16518)

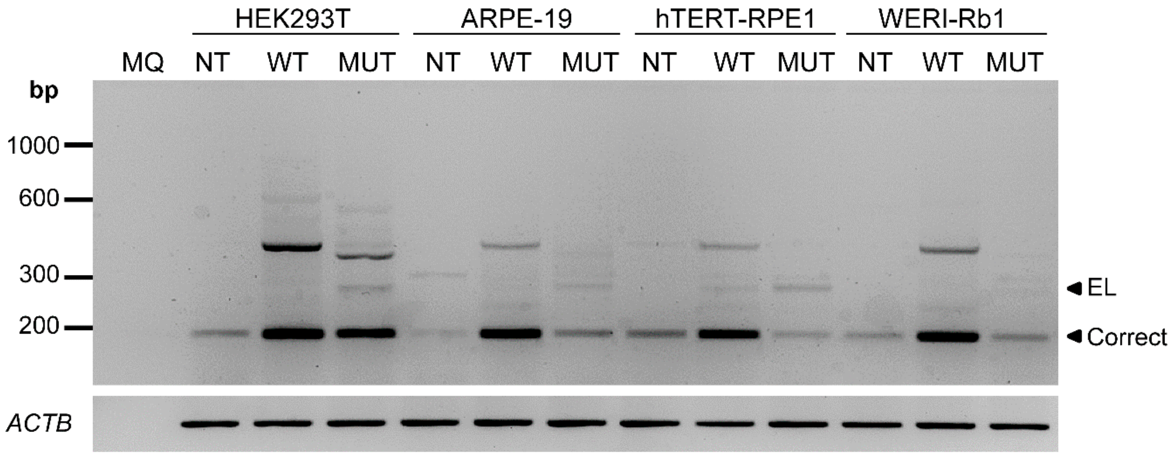

Figure S2. RT-PCR from exon 1 to 3 of the *RPE65* entry clones in different cell lines. A) Splice assays in HEK293T, ARPE-19, hTERT-RPE1 and Weri-Rb1. Representative electrophoresis gel (n=2) of the RT-PCR product after amplifying exon 1 to 3 of *RPE65* comparing wild-type (WT) and mutant (MUT) expression vectors. MQ: miliQ water; NT: non-transfected; EL: exon elongation; Correct: wild-type transcript; bp: base-pairs.
